# Timing Matters: Time of Day Impacts the Ergogenic Effects of Caffeine—A Narrative Review

**DOI:** 10.3390/nu16101421

**Published:** 2024-05-08

**Authors:** Ye Zhang, Weijun Yang, Yizhang Xue, Dingchun Hou, Songyue Chen, Zhiqin Xu, Sijia Peng, Haotian Zhao, Can Wang, Chang Liu

**Affiliations:** 1 Sport Coaching College, Beijing Sport University, Beijing 100084, China; 2 Institute of Population Research, Peking University, Beijing 100871, China; 3 Department of Bioengineering, University of California, Los Angeles, Los Angeles, CA 90095, USA; 4 School of Sport Science, Beijing Sport University, Beijing 100084, China; 5 National Engineering Research Center of Fruit and Vegetable Processing, College of Food Science and Nutritional Engineering, China Agricultural University, Beijing 100083, China; 6 Department of Physical Education, Jiangnan University, Wuxi 214122, China; 7 College of Food Science and Nutritional Engineering, China Agricultural University, Beijing 100083, China

**Keywords:** caffeine, ergogenic effects, circadian rhythms, sports performance, administration timing

## Abstract

Caffeine has attracted significant attention from researchers in the sports field due to its well-documented ergogenic effects across various athletic disciplines. As research on caffeine continues to progress, there has been a growing emphasis on evaluating caffeine dosage and administration methods. However, investigations into the optimal timing of caffeine intake remain limited. Therefore, this narrative review aimed to assess the ergogenic effects of caffeine administration at different times during the morning (06:00 to 10:00) and evening (16:00 to 21:00). The review findings suggest that circadian rhythms play a substantial role in influencing sports performance, potentially contributing to a decline in morning performance. Caffeine administration has demonstrated effectiveness in mitigating this phenomenon, resulting in ergogenic effects and performance enhancement, even comparable to nighttime levels. While the specific mechanisms by which caffeine regulates circadian rhythms and influences sports performance remain unclear, this review also explores the mechanisms underlying caffeine’s ergogenic effects, including the adenosine receptor blockade, increased muscle calcium release, and modulation of catecholamines. Additionally, the narrative review underscores caffeine’s indirect impact on circadian rhythms by enhancing responsiveness to light-induced phase shifts. Although the precise mechanisms through which caffeine improves morning performance declines via circadian rhythm regulation necessitate further investigations, it is noteworthy that the timing of caffeine administration significantly affects its ergogenic effects during exercise. This emphasizes the importance of considering caffeine intake timing in future research endeavors to optimize its ergogenic potential and elucidate its mechanisms.

## 1. Introduction

Caffeine (1,3,7-trimethylxanthine), a compound commonly present in various soft drinks and food items, originally sourced from coffee, serves as a widely utilized athletic performance enhancer among both active individuals and elite athletes [1]. Dating back to the first known study published in 1907, the performance-enhancing effects of caffeine have been extensively studied for over a century [2].

Due to its demonstrated ability to enhance performance in sports such as running, swimming, and cycling, caffeine was previously classified as a banned substance in sports from 1984 to 2004 and subject to strict prohibition. However, owing to concerns regarding its susceptibility to inadvertent ingestion, potential misuse, and athlete welfare, the World Anti-Doping Agency (WADA) ultimately opted to remove caffeine from the banned substances list, lifting the prohibition on 1 January 2004 [3]. Following these developments, interest in caffeine has surged, establishing it as one of the most well-established ergogenic aids with performance-enhancing effects across a variety of sports disciplines [4].

However, the specific factors governing its optimal utilization necessitate further investigation [5]. For instance, recent literature reviews have examined how dosage [6], administration method [7], and training context [8] affect the ergogenic effects of caffeine. Nonetheless, limited research has explored the influence of time of day on the ergogenic effects of caffeine consumption [9]. This review aims to address this gap in the current literature. Additionally, there exists an ongoing debate concerning the efficacy of caffeine as an ergogenic aid in enhancing exercise performance [10]. Several research studies have suggested that caffeine may not yield significant improvements in exercise performance [11]. 

Therefore, we hypothesize that the timing of caffeine consumption plays a pivotal role in determining its effectiveness in enhancing athletic performance. Notably, recent literature frequently highlights a decline in exercise performance during the morning hours, which can be effectively mitigated by caffeine supplementation to enhance exercise capacity [12]. Moreover, the influence of the circadian rhythm on caffeine’s effects is noteworthy. However, current research on these phenomena, including evidence and underlying mechanisms, is fragmented and lacks a unified framework for review. This review endeavors to bridge this gap by elucidating the intricate relationship between caffeine, circadian rhythms, and exercise performance, underscoring the necessity for further original experimental research in this domain.

## 2. Methods

This narrative review employed a literature search spanning articles published between 1980 and April 2024, sourced from databases including PubMed, Cochrane Library, Embase, Scopus, and Web of Science. The search aimed to encompass all studies examining the impact of caffeine on athletic performance during this period. Search terms included “Caffeine”, “Ergogenic effects”, and “Sports performance”, combined using the Boolean operators “AND” and “OR”. The database search was performed by authors YZ and WY. Subsequently, retrieved articles were exported to an Excel^®^ sheet, where duplicate entries were identified and removed. Any discrepancies were resolved through discussion between the lead reviewer and a third reviewer (CL).

## 3. Caffeine Has Ergogenic Effects

### 3.1. The Influence of Caffeine on Athletic Performance

Athletes across various sports disciplines, including strength athletes like weightlifters, endurance athletes such as marathon runners, cyclists, and triathletes, and competitive athletes, including handball, tennis, and volleyball players, frequently consume caffeine [11]. In a study analyzing 20,686 urine samples from elite athletes, it was found that 73.8% exhibited caffeine concentrations surpassing 0.1 μg·mL^−1^, indicating a prevalent consumption of caffeine before or during sports competition [13].

Research investigating the impact of caffeine intake on exercise performance has explored its effects across various exercise tests, including aerobic endurance performance [14], muscular endurance [15], 1 repetition maximum (1 RM) intensity [16], vertical jump height [17], isokinetic peak torque [18], and power output in different exercise modalities [19]. Additional systematic reviews have underscored the synergistic ergogenic effects of caffeine on the specific aspects of power-based sports [20], resistance exercises [21], and sport-specific endurance [22].

Following caffeine’s removal from the banned list of stimulants by WADA, its utilization among athletes has surged. Studies indicate that 67% of athletes experience enhanced sports performance following caffeine consumption, while 33% either report no change or may even suffer adverse effects [23]. This variability underscores the nuanced nature of caffeine’s impact, despite its confirmed synergistic potential. 

Current research suggests that caffeine’s efficacy in improving exercise performance may stem from one or a combination of the following mechanisms: (1) facilitation of calcium release in the sarcoplasmic reticulum [24]; (2) preservation of muscle glycogen through phosphodiesterase inhibition [25]; and (3) antagonism of the adenosine A1 and A2 receptors in the central nervous system [26].

Although the precise mechanism underlying caffeine’s ergogenic effects on exercise capacity remains elusive, various factors including dosage, duration of intake, withdrawal effects, habitual consumption, training level, and genetic predisposition all influence its efficacy [11]. Notably, the impact of caffeine on athletic performance varies significantly based on these factors.

Regarding dosage, the performance-enhancing effects of low-dose caffeine intake (≤3 mg·kg^−1^) primarily result from its direct modulation of the central nervous system [6]. Conversely, higher doses (6–9 mg·kg^−1^) during aerobic exercise can alter substrate oxidative flux, shifting towards increased lipid utilization while preserving muscle glycogen stores [27]. This mechanism contributes to improvements observed in aerobic performance tests [28]. Furthermore, higher caffeine doses may induce additional performance benefits by enhancing calcium release from the sarcoplasmic reticulum [29].

The timing of caffeine ingestion also significantly influences its ergogenic effects. Whether in low, medium, or high doses, caffeine alone typically reaches peak plasma concentration 30 to 90 minutes post-ingestion [30]. However, research suggests that the “optimal” timing for caffeine consumption is not solely correlated with the peak plasma concentration after ingestion but encompasses a broader “window” of possibilities [31]. Additionally, when combined with carbohydrates, caffeine’s plasma concentration profile becomes more gradual and exhibits fewer peaks, leading to enhanced effects on cycling performance [32].

Another crucial factor to consider is the withdrawal effect of caffeine, which is present in nearly all caffeine-related studies [33]. Typically, participants abstain from caffeine intake for a period of 6 to 10 days between experiments to mitigate the influence of caffeine withdrawal symptoms [34]. While caffeine deprivation is essential for the experimental design, sudden cessation of caffeine consumption can lead to various adverse effects due to its ubiquitous presence in a variety of everyday foods and beverages. These withdrawal symptoms may include depression, headaches, decreased alertness and productivity, increased drowsiness or fatigue, stiffness, nausea, and irritability [35]. Importantly, these side effects can impact athletic performance, and any subsequent performance benefits observed upon reintroduction of caffeine may stem from the alleviation of these withdrawal effects rather than a direct enhancement in performance [36]. Therefore, it is imperative to consider and potentially eliminate these confounding factors to gain a clearer understanding of the true effects of caffeine on exercise performance.

The degree of training plays a crucial role in influencing the ergogenic effects of caffeine. While the precise mechanisms remain elusive and occasionally contradictory, existing findings unequivocally underscore the significance of training level. Research indicates that trained individuals often exhibit an enhanced neuromuscular action potential, potentially resulting in greater performance enhancements from caffeine consumption compared to their untrained counterparts [37]. Conversely, investigations reveal that trained individuals possess elevated concentrations of adenosine A_2A_ receptors relative to untrained individuals, necessitating higher caffeine intake for comparable stimulatory effects [38]. Nevertheless, both lines of inquiry converge on the pivotal observation that training level significantly impacts caffeine’s ergogenic effects.

### 3.2. The Mechanisms of Caffeine as an Ergogenic Aid

Caffeine is believed to enhance performance in various sports and exercise modalities through three primary mechanisms, namely the adenosine receptor blockade, increased muscle calcium release, and effects on catecholamines. However, despite the mounting evidence indicating caffeine’s multifaceted effects, including its potential to enhance fat metabolism and induce the release of irisin from muscles, and the subsequent browning of white fat to release energy, there is currently no direct evidence linking these effects to improvements in exercise capacity [39,40].

Blocking adenosine: 

The chemical structure of caffeine bears resemblance to that of adenosine, a naturally occurring molecule in the body [41]. Adenosine binds to purinergic G protein-coupled receptors, specifically adenosine receptors A_1_ and A_2A_, which are the targets of corresponding endogenous ligands in the brain [42]. This binding typically results in sensations of fatigue, drowsiness, and potentially pain, while also modulating the release of neurotransmitters such as dopamine and glutamate [43]. Increased levels of adenosine correlate with heightened fatigue [44].

Given the structural similarity between caffeine and adenosine, caffeine functions by antagonizing adenosine receptors, thereby impeding adenosine’s ability to bind to these receptors [45]. Consequently, this inhibition diminishes the sensations of fatigue and pain, induces euphoria, and elevates the heart rate [46]. This phenomenon is commonly referred to as the central effect of caffeine, denoting its impact on the brain.

Caffeine augments calcium release in muscle cells:

Caffeine enhances the release of calcium within the muscle cells through the modulation of calcium ion channels present in the cell membranes, pivotal for muscle contraction [16]. Intracellular Ca^2+^ signals are typically amplified via the discharge of significant quantities of calcium from the endoplasmic reticulum [47]. This phenomenon, known as calcium-induced calcium release, is facilitated by the activation of the endoplasmic reticulum calcium channel, the ryanodine receptor (RyR) [48]. RyR activation is driven by both cytoplasmic calcium and endoplasmic reticulum calcium levels [49].

Caffeine serves to sensitize RyR to Ca^2+^, thereby promoting endoplasmic reticulum Ca^2+^ release even under basal cytoplasmic Ca^2+^ conditions [50]. This augmented influx of calcium ions triggers muscle cell contraction, subsequently enhancing both the strength and endurance of muscle contractions [51]. Consequently, the physiological effects of caffeine culminate in heightened calcium release within muscle cells, thereby augmenting muscle functionality and performance.

Caffeine affects catecholamine levels:

Catecholamines represent a group of phenylethylamine compounds characterized by a catechol core structure [52]. They serve as stress-related sympathomimetic hormones produced by the adrenal glands, eliciting the “Fight or Flight” response [53]. Among the primary catecholamines are epinephrine (adrenaline), norepinephrine (noradrenaline), and dopamine, synthesized from phenylalanine and tyrosine [54]. Norepinephrine is extensively distributed in the central nervous system and is abundant, while epinephrine occurs in lesser quantities [55]. Dopamine is primarily concentrated in the extrapyramidal system and functions as a neurotransmitter [56]. All these compounds serve as pivotal classic adrenergic receptor agonists.

Caffeine induces a rise in catecholamine levels, particularly epinephrine [57]. Elevated catecholamine levels can promote lipolysis, leading to increased breakdown of fats [58]. Under conducive circumstances with ample fatty acids available, these fats can be utilized as energy substrates during physical exertion [59]. Increased reliance on fat oxidation may potentially reduce glycogen breakdown, the body’s stored form of carbohydrates, thereby preserving it for later use and potentially delaying the onset of fatigue or exhaustion, ultimately enhancing performance [60].

## 4. Circadian Rhythm on Sport Performance

### 4.1. Circadian Rhythms Affects Human Physiology

Circadian rhythms are innate biological oscillations that repeat approximately every 24 h, with an average duration of 24.09 h for women and 24.19 h for men [61]. These rhythms encompass any internally driven process within an organism that responds to, or is influenced by, external environmental cues [62]. Regulated by the biological clock, circadian rhythms synchronize biological functions to occur optimally for an individual’s health [63]. Recent research indicates that circadian rhythms are entrainable, meaning they can be reset by external stimuli such as light and heat [64]. Changes in circadian rhythm are closely linked to key phase markers, including pineal secretion of melatonin, minimum core body temperature, and plasma cortisol levels [65].

In recent years, there has been increasing evidence linking sports performance, encompassing aerobic and anaerobic fitness as well as fine and gross motor skills, to circadian rhythms [66]. The suprachiasmatic nucleus, located in the hypothalamus, serves as the central pacemaker for these rhythms. It receives direct input from the retina, synchronizing with the solar cycle [67]. This synchronization, facilitated by the retinal-hypothalamic pathway, regulates the suprachiasmatic nucleus’ response to solar time and subsequently governs sleep-wake cycles, thereby coordinating various daily biological rhythms such as temperature fluctuations, hormone secretion, and neural activation [68].

Several factors contribute to variations in athletic performance across different times of the day, reflecting the influence of circadian rhythms. These factors include reduced flexibility in the morning, disparities in nutritional intake between morning and evening, individual preferences for training times, inadequate recovery time from sleep inertia, variations in physiological responses among individuals, differences in motivation and anticipated effects, as well as variations in rest periods between assessments. These variables collectively help elucidate the impact of circadian rhythms on athletic performance [69].

Numerous factors, both the internal physiological and external environmental, influence the circadian rhythm of bodily performance throughout the day. External elements such as ambient temperature and environmental conditions can significantly impact physical and psychological arousal. Additionally, an individual’s biological rhythms and their ability to synchronize with external rhythms play a crucial role. Lifestyle choices, including activity timing, sleep patterns, and the capacity to manage sleep inertia and sleep deprivation, further contribute to this dynamic [70].

Studies indicate a strong correlation between the common morning performance nadirs and evening peaks in exercise and circadian rhythms [71]. This correlation may be attributed to the pivotal role of core body temperature, which closely aligns with circadian rhythms. Human body temperature follows a diurnal pattern, rising during the day and falling at night, in coordination with the circadian rhythm. This fluctuation interacts with the molecular clock and genes controlled by biological clocks, including immune-related genes [72]. The circadian rhythm of body temperature is a key feature of the central biological clock, which arises from complex biological processes. This rhythm may contribute to synchronizing circadian clocks in peripheral tissues, ensuring proper immune system function [73].

Thermoregulatory circadian rhythms involve neuromodulation, with the brain’s circadian clock region communicating with other tissues via neural pathways to regulate body temperature changes [74]. Hormonal regulation also plays a role, with hormones such as cortisol exhibiting fluctuations in secretion levels across the circadian cycle, influencing body temperature [75]. Additionally, metabolic rate changes affect body temperature [76]. These regulatory mechanisms enable the human body to maintain an appropriate body temperature throughout the day, supporting overall physiological function and health.

As core temperature rises, there is a tendency for increased carbohydrate utilization and decreased reliance on fat as an energy source [77]. Furthermore, elevated core temperature may enhance the actin–myosin cross-bridge mechanics within the musculoskeletal system, leading to peak body temperature and optimal performance typically occurring in the evening [78]. Furthermore, the sarcoplasmic reticulum increases calcium release, enhances calcium ion binding to actin–myosin complexes, and augments myosin’s ATPase activity, ultimately resulting in elevated levels of exercise performance [79].

Likewise, circadian rhythms are intricately linked to fatigue, non-functional overtraining, decision-making during exercise, hormonal fluctuations, immune system activity, nutritional supplementation, homeostasis, and numerous other factors that collectively impact athlete performance. While findings from various studies may differ, they consistently highlight a central theme, that optimal sports performance typically occurs in the afternoon, coinciding with the peak of core body temperature. The timing of exercise during the day significantly influences the attainment of peak physical performance [71].

### 4.2. Circadian Rhythms Affects Sport Performance

Circadian rhythms play a fundamental role in humans’ physiological and behavioral processes [80]. Specifically, during exercise, they have been demonstrated to significantly influence success and failure in various endeavors, including competitive sports [81]. The rhythmic activity and performance of an athlete’s body profoundly impact their performance in intense competitions [82]. Physical activities and training schedules should be aligned with an athlete’s circadian rhythm type and peak performance times to optimize performance and achievement [66]. The precision and temporal variability of individual peak performance may be influenced by several factors, including the circadian clock, jet lag, light exposure, time of day, hormonal fluctuations, and body temperature [83]. Circadian rhythms affecting mood, ultimate athletic performance, core body temperature, and alertness are influenced not only by sleep patterns but also by circadian changes in hormones such as cortisol, testosterone, and melatonin [84].

Athletic performance is influenced by circadian rhythms, with a better performance typically observed in the afternoon compared to the early morning [70]. A systematic review and meta-analysis focusing on resistance training at different times of the day revealed distinct intensity variations between morning and evening sessions, with greater intensity observed in the evening, indicating peak muscle capacity (e.g., strength) during nighttime [85]. Similarly, a study examining repeated sprints in the morning and afternoon found significantly higher power output in afternoon sprints (23.3 ± 1 W·kg^−1^) compared to morning sprints (21.2 ± 1 W·kg^−1^, *p* < 0.05) [86]. Investigations testing the maximum torque of knee extensor muscles at various time points throughout the day demonstrated significantly lower torque production at 06:00 and 10:00 (approximately 90% of maximum) compared to 18:00 (approximately 99% of maximum, *p* < 0.05) [87].

Assessing the impact of the time of day on performance, the findings regarding the circadian rhythm, and hormonal and metabolic responses, during a 1000 m cycling time trial revealed that completion time was shorter in the evening (88.2 ± 8.7 s) compared to the morning (94.7 ± 10.9 s, *p* < 0.05). Moreover, total testosterone, free testosterone, insulin, and cortisol concentrations were notably higher in the morning (+31%, +22%, +60%, and +26%, respectively, *p* < 0.05), whereas the growth hormone was doubled in the evening (*p* < 0.05). Morning blood glucose levels decreased by approximately 11% (*p* < 0.05). Epinephrine, norepinephrine, lactate, and glucagon levels remained similar between morning and evening trials (*p* > 0.05), although the norepinephrine response to exercise increased in the morning (+46%, *p* < 0.05), accompanied by a fold increase in 5-glucose response [88].

### 4.3. The Mechanism by Which Circadian Rhythms Affects Sport Performance Remain Unclear

Research has investigated the effects of resistance training conducted at specific times of the day on muscle hypertrophy, protein phosphorylation, hormone concentrations, and neuromuscular performance. In a study, 25 previously untrained men were randomly assigned to morning, afternoon, and control groups. Both the morning and afternoon groups underwent hypertrophy resistance training, with 22 sessions conducted between 07:30 and 08:30, and between 16:00 and 17:00, respectively, over 11 weeks. Results indicated a significant increase in voluntary muscle strength in both the morning and afternoon training groups, by 16.9% and 15.2%, respectively. Muscle hypertrophy incidence was 8.8% in the morning group and 11.9% in the afternoon group (*p* < 0.001), with muscle fiber cross-sectional area levels at 21% and 18% respectively (*p* < 0.01) [89]. 

To elucidate the molecular biological mechanisms underlying this phenomenon, research findings indicate that under pre-training conditions, post-load phosphorylation of ribosomal protein S6 kinase beta-1 (S6K1), also known as P70-S6 Kinase 1 (p70S6^Thr421/Ser424^) increased irrespective of the time of day. p70S6^Thr421/Ser424^ is a downstream target of the mammalian target of rapamycin (mTOR) signaling, specifically mTORC1, an mTOR-containing complex characterized by the inclusion of Raptor rather than Rictor (mTORC2). Physical exercise triggers protein synthesis by activating p70S6^Thr421/Ser424^ through phosphorylation, a process dependent on the mTOR, particularly mTORC1. This mechanism has been substantiated through the use of rapamycin, an mTOR inhibitor, which impedes muscle mass gains despite increased loading such as exercise. Additionally, exercise has been observed to elevate the insulin-like growth factor 1 (IGF-1) levels in muscles, thereby initiating the IGF-1/Phosphoinositide 3-kinase (PI3K)/Protein kinase B (Akt)/p70S6^Thr421/Ser424^ signaling pathway. This cascade enhances protein synthesis essential for muscle growth and development [90].

However, in the morning group, this phosphorylation significantly increased only after the training period (*p* < 0.05). Phosphorylation of the ribosomal protein S6 (rpS6) and p38 mitogen-activated protein kinases (p38MAPK) increased significantly both before and after training, regardless of the time of day (*p* < 0.05). The phosphorylation of rpS6 and p38MAPK plays a crucial role in cellular signaling and regulation, particularly in response to various stimuli including growth factors, stress, and exercise. Phosphorylation of rpS6 is typically associated with the activation of the mTORC1 pathway. Activation of rpS6 through phosphorylation promotes ribosomal biogenesis and protein translation, ultimately leading to increased protein synthesis and cellular growth. This process is essential for muscle hypertrophy and adaptation to exercise, as well as other cellular processes such as cell proliferation and survival [91].

On the other hand, p38^MAPK^ is a stress-activated protein kinase involved in the cellular response to various stressors, including oxidative stress, inflammatory cytokines, and mechanical stress such as exercise. Phosphorylation of p38^MAPK^ regulates a wide range of cellular processes, including gene expression, cell cycle regulation, apoptosis, and cellular differentiation. In the context of exercise, the phosphorylation of p38^MAPK^ is associated with the activation of various signaling pathways that mediate adaptations to exercise-induced stress, such as muscle repair, mitochondrial biogenesis, and the regulation of inflammatory responses [92].

Conversely, the phosphorylation of p70S6^Thr389^, eEF2, and Erk1/2 remained unchanged at all-time points. Indeed, the p70S6 kinase (p70S6^Thr389^), encoded by the RPS6KB1 gene, is a crucial downstream effector of the PI3K/mTOR pathway. This pathway plays a pivotal role in regulating various cellular processes, including cell survival, proliferation, and metabolism. The activation of p70S6^Thr389^ is mediated by phosphorylation at multiple sites, including threonine 389 (Thr389), which is a key regulatory phosphorylation site. Upon activation, p70S6K phosphorylates ribosomal protein S6 (RPS6) and other downstream targets involved in protein synthesis and cell growth. The PI3K/mTOR signaling pathway is indeed a central regulator of translation, controlling the synthesis of proteins involved in various cellular processes. Disruptions in this pathway have been implicated in numerous pathological conditions, including cancer, rendering it a significant target for therapeutic intervention [92,93].

Elongation Factor 2 (eEF2) is a protein involved in the process of translation, specifically during the elongation phase. Translation is the process by which messenger RNA is decoded by ribosomes to produce proteins. During translation elongation, eEF2 facilitates the translocation of the ribosome along the messenger RNA molecule, allowing the ribosome to move from one codon to the next and enabling the addition of amino acids to the growing polypeptide chain. The activity of eEF2 is regulated by phosphorylation. Phosphorylation of eEF2 by the eEF2 kinase (eEF2K) inhibits its function, slowing down protein synthesis. This phosphorylation is often regulated by various signaling pathways in response to cellular conditions, such as nutrient availability, energy status, and stress. The regulation of eEF2 activity is crucial for controlling the rate of protein synthesis and maintaining cellular homeostasis. Dysregulation of eEF2 phosphorylation can impact various cellular processes and has been implicated in diseases such as cancer and neurodegenerative disorders [94].

Extracellular Signal-Regulated Kinases 1 and 2 (ERK1/2) are members of the mitogen-activated protein kinase (MAPK) family and play essential roles in cell signaling. These kinases are activated by various extracellular stimuli, including growth factors, cytokines, and hormones, and are involved in regulating diverse cellular processes such as proliferation, differentiation, survival, and migration. Activation of ERK1/2 typically occurs through a phosphorylation cascade. Upon stimulation of cell surface receptors, such as receptor tyrosine kinases or G protein-coupled receptors, intracellular signaling pathways are activated, leading to the activation of a kinase cascade. Once activated, ERK1/2 translocate into the nucleus where they phosphorylate various transcription factors and other nuclear targets, thereby modulating gene expression and influencing cellular responses. ERK1 and ERK2 are highly homologous and share similar functions, but they may also exhibit distinct roles depending on the cellular context. Dysregulation of the ERK1/2 signaling pathway has been implicated in various diseases, including cancer, where aberrant activation of ERK1/2 can promote uncontrolled cell proliferation and tumor progression. Consequently, targeting the ERK1/2 pathway has been explored as a therapeutic strategy for cancer treatment [95].

Meanwhile, resting cortisol levels significantly decreased from pre- to post-training in all three groups (*p* < 0.05). These findings indicate that certain parameters of skeletal muscle signaling may undergo time-dependent adaptations. However, the precise molecular biological mechanisms responsible for these adaptations remain unclear [89].

## 5. Caffeine Alleviates Morning Performance Decline

### 5.1. Caffeine Mitigates Declines in Morning Performance Induced by Sleep Deprivation

Given that athletes frequently engage in training and competition during early morning hours, there is a growing interest in strategies to mitigate the decline in morning performance [96]. According to a recent study, participants were subjected to sleep deprivation (3–5 h of sleep) or served as controls (with 7–9 h of sleep), followed by supplementation with or without caffeine. They then performed repetitive rugby passing skills. Placebo sleep deprivation led to a significant decrease in skill performance accuracy for both dominant and non-dominant passers (*p* < 0.001). However, the study results indicated that there was no decline in skill performance at caffeine doses of 1 or 5 mg/kg, and the effects of the two doses were not significantly different. Acute sleep deprivation was found to impair the performance of simple repetitive skills in elite athletes. A single dose of caffeine may help alleviate the decline in skill performance under sleep-deprived conditions, such as during trans meridian travel. Additionally, low doses of caffeine appear to be as effective as high doses in this regard [97].

Other experiments involving sleep deprivation have also demonstrated the efficacy of three doses of 2 mg/kg caffeine in assisting 12 recreational runners in completing a run to failure at 75% of their final velocity around a 400 m outdoor track and field track in the Vameval test, as well as in performing correct detection and reaction time tasks. The Vameval test is a progressive shuttle run test commonly used to assess aerobic fitness and maximal aerobic speed. During the Vameval test, participants run back and forth between two markers placed 20 m apart. The test begins at a relatively low speed, with the pace gradually increasing at predetermined intervals signaled by audio beeps. Participants must reach the marker before each beep to maintain the required pace. As the test progresses, the intervals between beeps shorten, requiring participants to run faster. The test continues until the participant can no longer maintain the pace or fails to reach the marker before the beep on two consecutive occasions. The Vameval test provides an estimate of the participant’s maximal aerobic speed, which is a valuable measure of cardiovascular fitness and endurance capacity. It is commonly used in sports training, physical education, and research settings to assess aerobic performance and monitor changes over time [98].

Complete sleep deprivation conditions significantly impaired running performance, reaction time, and correct detection compared to controls. Conversely, caffeine intake improved running performance to exhaustion by 5.2% in the control group (*p* < 0.001) and by 8.9% in the complete sleep deprivation group (*p* < 0.001). Thus, repeated low-dose caffeine intake emerges as an effective strategy for mitigating the adverse effects of complete sleep deprivation on both physical and cognitive performance [99]. 

Similarly, findings from a study involving 13 healthy male physical education students who underwent 36 h of sleep deprivation revealed that caffeine intake enhanced peak values measured in the Wingate test 30 s after complete sleep deprivation (*p* < 0.001), squat jumps (*p* < 0.001), and reduced reaction time (*p* < 0.001) [100]. The Wingate test is a widely used anaerobic exercise test that involves a short but intense burst of maximal effort and is typically performed on a cycle ergometer. During the test, the participant pedals as fast as possible against a predetermined resistance for a duration of around 30 s. The objective is to generate maximal power output during this brief period. The Wingate test provides valuable insights into an individual’s anaerobic capacity, muscular strength, and power. It is commonly employed in sports science, exercise physiology, and clinical settings to assess athletic performance, monitor training progress, and evaluate interventions aimed at improving anaerobic performance [101].

### 5.2. Caffeine Alleviates Morning Performance Declines

The decline in morning performance, a prevalent issue in sports and competitive activities, has garnered increasing attention and become a research focus within the sports community in recent years. Caffeine has emerged as an effective intervention for mitigating the decline in morning performance. A study involving twelve highly resistance-trained men demonstrated that caffeine consumption counteracted morning neuromuscular decline, thereby enhancing performance to levels akin to those observed in afternoon trials. In the afternoon placebo trial conducted at 18:00, dynamic muscle strength and power output exhibited significant improvement compared to the morning placebo treatment at 10:00 (3.0–7.5%; *p* ≤ 0.05). Conversely, during the morning caffeine ingestion period (10:00 a.m.) at a dosage of 3 mg kg^−1^, muscle strength and power output surpassed levels observed in the morning placebo treatment (4.6–5.7%; *p* ≤ 0.05), except for blood pressure velocity at a 1 ms^−1^ load (*p* = 0.06). The isometric electrically evoked strength of the right knee and norepinephrine levels (serving as a surrogate measure of maximal muscle sympathetic nerve activation) were notably higher during the morning caffeine trial compared to the morning placebo trial (14.6% and 96.8%, respectively; *p* ≤ 0.05). These findings, alongside data from electrical stimulation and norepinephrine assessments, suggest that caffeine holds the capability to augment neuromuscular performance and exert a direct influence on muscle function [102].

Similarly, thirteen resistance-trained men engaged in bench press and full squat exercises at maximal velocity, subject to four incremental loads (25%, 50%, 75%, and 90% of one repetition maximum). These trials were conducted at 60 minutes’ post ingestion of either 6 mg kg^−1^ caffeine or a placebo. Two trials occurred in the morning and two in the afternoon, with each pair being separated by 36–48 h. Measurements of the tympanic membrane temperature, plasma caffeine concentration, and side effects were taken. In the control group receiving no caffeine, tympanic membrane temperatures were significantly lower in the morning compared to the afternoon (36.7 ± 0.4 and 37.0 ± 0.5 °C, respectively; *p* < 0.05). Notably, at loads of 25%, 50%, and 75% of one repetition maximum in the full squat exercise, morning caffeine consumption resulted in significantly higher values (5.4–8.1%; *p* < 0.05) compared to the non-caffeine group. Conversely, during the afternoon trials, caffeine ingestion failed to enhance propulsive velocity at any load for either bench press or full squat. These findings suggest that moderate caffeine intake may counteract the decline in muscle contraction rate observed in the morning across various loads, with caffeine’s effects being more pronounced on lower body muscle tissue. Moreover, evening caffeine consumption appears to exert minimal impact on neuromuscular performance [103].

A further investigation evaluating the impact of caffeine supplementation at varying times of day on cycling performance revealed findings from twenty male participants (mean age: 25 years; peak oxygen consumption: 57 mL·kg^−1^·min^−1^). These participants underwent two familiarization trials and four experimental trials, featuring a computer-simulated 3 km bicycle time trial. The experimental setup involved combinations of morning and evening sessions, alongside either 6 mg/kg caffeine or a placebo. Results indicated that the trained athletes exhibited heightened caffeine-induced performance improvements in both morning (2.3% ± 1.7%) and evening (1.4% ± 1.1%) sessions compared to the placebo. Additionally, it was observed that untrained individuals experienced more pronounced performance enhancements from caffeine during evening sessions compared to their trained counterparts (5.5% ± 4.3%; 1.0% ± 1.7%, respectively) [104].

The study provided further evidence supporting previous findings that caffeine enhances morning performance, especially among trained individuals. While the specific mechanisms underlying this morning performance advantage with caffeine remain uncertain, it is speculated to be associated with physiological factors such as variations in caffeine metabolism rates and somatic control. Additionally, the study revealed differential responses to caffeine based on training status, with untrained individuals showing a more favorable response in the evening. However, further investigation into the precise physiological mechanisms driving these responses is warranted. Despite limitations such as sample size and fasting durations, the findings suggest that caffeine supplementation may be advantageous for morning competitions but may have less pronounced effects in the evening, particularly for trained individuals. Given the potential impact on sleep, athletes are advised to assess their individual responses to evening caffeine intake before use. Further research exploring external factors influencing individual responses to caffeine supplementation is necessary to optimize performance outcomes [104]. Studies have also explored the synergistic effects of caffeine consumption during the Wingate test on diurnal variations in mood state, simple reaction time, and muscle strength. Twelve elite judo athletes (mean age: 21.1 ± 1.2 years; weight: 83.8 ± 8.2 kg; height: 1.8 ± 0.6 m) took part in the investigation. Emotional states, simple reaction times, as well as peak and average power during the Wingate test were assessed across four separate sessions in a randomized design. Findings revealed heightened levels of anxiety, energy, and fatigue, alongside decreased simple reaction times following caffeine intake at 07:00 and 17:00 compared to those administered a placebo. Additionally, the study observed a reduction in the diurnal variation of peak power and mean power following caffeine ingestion compared to the control group, with performance enhancements detected solely at 07:00 (*p* < 0.05). These results suggest that consuming caffeine in the morning may mitigate diurnal fluctuations in short-term maximal performance [100,105].

A recent study examined the impact of acute caffeine consumption on diurnal variations in neuromuscular performance among women engaged in resistance training. Fifteen resistance-trained women took part in the investigation. Participants received acute doses of caffeine (3 mg/kg) or a placebo between 09:00 to 11:00 and/or 17:00 to 19:00, and the study evaluated neuromuscular performance, maximal strength, and strength endurance. The findings indicate that elevated morning caffeine intake significantly enhanced countermovement jump height performance by 3.1%, restoring it to levels observed in the afternoon. However, this effect was not observed in peak bench press throw velocity, lower body and upper body maximal strength, or strength endurance performance. Furthermore, women undergoing resistance training exhibited superior lower- and upper-body ballistic performance in the afternoon compared to the morning, with high caffeine intake proving effective solely in increasing the countermovement jump height [106].

A study comparing the impact of acute morning and evening caffeine supplementation (3 mg/kg) on performance-related parameters in basketball players involved 11 nationally ranked junior male basketball athletes who underwent in-field physical fitness testing on four separate occasions. Results suggest that the effects of caffeine supplementation on basketball-specific performance-related parameters are contingent upon the timing of ingestion in elite adolescent basketball players. Specifically, morning (10:00) caffeine intake led to marginal enhancements in vertical jump, change in direction, 20 m linear sprint, and repeated sprints compared to the morning (10:00) placebo intake. Similarly, evening (21:00) caffeine intake versus evening (21:00) placebo intake resulted in modest differences across all performance-related variables. Notably, the vertical jump height and 20 m sprint speed were compromised in the morning compared to the evening, but such disparities could be mitigated by morning caffeine consumption. It is advisable for basketball practitioners to administer caffeine exclusively in the morning to enhance vertical jump, sprinting, and change of direction performance. However, no additional performance benefits were observed when caffeine was consumed in the morning compared to evening ingestion [107].

### 5.3. Exploring the Heterogeneity of Caffeine’s Impact on Morning Performance Decrement

While a considerable body of research supports the efficacy of caffeine in mitigating morning performance declines, the timing of caffeine consumption appears to influence its effectiveness. Specifically, consuming caffeine in the morning yields superior improvements in athletic performance compared to evening intake. Nevertheless, certain studies have indicated that the timing of caffeine consumption throughout the day may not significantly impact its performance-enhancing effects.

In a study involving thirteen physically active men, participants underwent repeated sprint ability tests (10 × 6 s cycle sprints with 30 s rest) under the following four conditions: morning caffeine, morning caffeine placebo, afternoon caffeine, and afternoon placebo. The findings revealed that total work (+8%, d = 0.2, small), peak power (+6%, d = 0.2), and anaerobic power reserve (+9%, d = 0.2) were significantly higher in the afternoon compared to the morning. However, there were no discernible differences in physiological responses between the caffeine and placebo conditions. Notably, performance on the repeated sprints was influenced by the time of day, with performance being lower in the morning compared to the afternoon. Nevertheless, caffeine supplementation failed to counteract the decline in morning performance or enhance performance in the afternoon [108].

## 6. Future Perspectives

### 6.1. Considerations for Timing Factors in Future Caffeine Research

In future research endeavors, it may be imperative for researchers to carefully consider the timing of caffeine ingestion to conduct studies that are more scientifically robust. Caffeine, being a significant sports nutrition supplement, has consistently garnered considerable attention [109]. Early investigations have substantially propelled research advancements in this domain and contributed to the widespread utilization of caffeine in daily practices [110,111,112,113,114]. While the majority of studies have indicated caffeine’s performance-enhancing effects across various athletic disciplines, some have yielded inconclusive results regarding its efficacy in certain performance metrics [115]. It remains unclear whether such discrepancies are linked to variations in administration timing. Hence, in forthcoming studies, researchers may benefit from explicitly specifying the precise timing of caffeine dosage during experiments.

Likewise, while many review articles and meta-analyses comprehensively evaluate caffeine’s ergogenic effects across diverse parameters, they often overlook the element of administration timing, potentially due to a paucity of relevant literature. It would be advantageous to incorporate discussions regarding the administration timing of caffeine, such as morning versus evening dosing, into these syntheses. Despite the significant scientific contributions of previous research in elucidating the role of caffeine in the realm of sports, there remains ample room for further refinement and depth in future investigations [116,117,118,119,120]. Consequently, we advocate for an increased emphasis on administration timing in future clinical experimental studies and review articles to advance our understanding of this critical aspect of caffeine supplementation.

### 6.2. Mechanisms of Caffeine in Alleviating Morning Performance Decline Require Investigation

While we have provided an overview and elucidated the impact of circadian rhythms on exercise performance and the role of caffeine in mitigating declines in morning performance, the specific mechanisms through which caffeine modulates circadian rhythms during exercise remain largely unknown. As this article serves as a review paper, we lack experimental evidence to validate the molecular biological mechanisms underlying this phenomenon. Therefore, we aim to present a simplified explanation of caffeine’s role in exercise and its general effects on regulating circadian rhythms. We hope to encourage further investigation by professional researchers to shed light on this intriguing topic and further advocate for caffeine as a valuable nutritional supplement in sports.

### 6.3. Mechanisms of Caffeine in Regulating Circadian Rhythms

There is currently inadequate evidence to assert that caffeine, in isolation and without concurrent exposure to ambient light, directly impacts circadian rhythms. Nevertheless, caffeine is likely to indirectly influence the body’s circadian rhythm by augmenting the organism’s responsiveness to light-induced phase shifts [44].

The central circadian pacemaker, situated within the suprachiasmatic nucleus (SCN) of the hypothalamus, serves as the primary regulator of daily physiological and behavioral changes, including sleep patterns [121]. Light, being the most potent synchronizer of the circadian clock, reaches the SCN via the retinohypothalamic tract, prompting the release of glutamate from its nerve terminals [122]. This glutamate release leads to heightened neuronal activity within the SCN [123]. Adenosine, which accumulates during prolonged wakefulness and typically inhibits neuronal activity, alongside serotonin, significantly influences SCN neuronal function [124].

Adenosine acts within the SCN’s photoreceptive ventrolateral zone, where it inhibits activity in the retinohypothalamic tract [125]. Stimulation of A_1_ receptors impedes excitatory synaptic transmission by obstructing intracellular Ca^2+^ accumulation [126]. Consequently, at a behavioral level, the administration of adenosine agonists diminishes light-induced phase shifts [127]. However, caffeine, acting as an A_1_ receptor antagonist, effectively counters this process [128]. Acute caffeine consumption amplifies the body’s response to light-induced phase shifts [129]. Similarly, sleep deprivation may diminish the circadian pacemaker’s phase-shifting capability by attenuating the intensity of light signals in the retinohypothalamic tract, due to inhibited glutamate release, and caffeine mitigates this inhibitory effect by modulating adenosinergic mechanisms [122].

In addition, numerous studies have indicated that the biological clock plays a significant role in influencing exercise performance, particularly in the context of a decline in morning performance [130]. Chronotype refers to an individual’s inherent preference for activity and alertness at specific times of the day, categorizing individuals into morning types, evening types, or intermediate types based on their peak performance periods [131]. Considering that many of these studies primarily involve elite athletes or adolescents, who are more inclined towards being “nocturnal types”, their performance may exhibit temporal variations. Specifically, if a majority of participants in these studies demonstrate a tendency towards late sleep patterns, they might naturally perform better in the evening compared to the morning. However, no studies have directly and conclusively established that caffeine intake can potentially enhance performance by simulating physiological states associated with the evening.

Nevertheless, it remains unclear whether caffeine’s ability to mitigate the decline in morning performance is influenced by the circadian clock. For individuals with evening chronotypes, morning caffeine consumption may mimic physiological states associated with the evening, potentially leading to improved performance [132]. To explore this hypothesis, future research should investigate the interaction between sleep chronotype and the effectiveness of caffeine intake or administration in enhancing exercise performance. By accounting for individual differences in circadian rhythms, researchers may uncover how caffeine impacts individuals with varying chronotypes and whether its effects are contingent upon the time of day. Such investigations could yield valuable insights into optimizing caffeine strategies for athletes across different sleep chronotypes.

The impact of caffeine on melatonin also represents a significant mechanism in its regulation of circadian rhythms. Melatonin, secreted by the pineal gland primarily during nighttime, plays a pivotal role in modulating the biological clock and facilitating sleep. Studies indicate that caffeine can disrupt pineal gland function, leading to inhibition of melatonin secretion. Consequently, caffeine consumption markedly diminishes melatonin levels on the day of intake and triggers early gene expression in the SCN, the central circadian pacemaker [133].

Additionally, caffeine has been shown to modulate the phase of electrical activity rhythm in the SCN, potentially advancing or delaying its onset, and extending its duration [134]. Moreover, caffeine intake delays the initiation of sleep phases, which represent distinct stages in the sleep cycle. Melatonin promotes the onset of these phases, with secretion peaking during nighttime. Disruption of this natural circadian mechanism by caffeine can delay melatonin’s peak secretion, resulting in difficulties falling asleep and disturbances in sleep cycles [134,135].

Furthermore, chronic and excessive caffeine consumption may lead to diminished sleep duration and alterations in sleep architecture. The stimulatory effects of caffeine can contribute to difficulties initiating sleep, increased dreaming, and reduced deep sleep. These sleep disruptions compromise the quality and restorative function of sleep, subsequently impacting daytime cognitive function, memory, and emotional stability [136].

These mechanisms highlight the significant role of caffeine in modulating circadian rhythms through its interaction with melatonin. However, the specific contribution of these mechanisms to alleviating these declines in morning performance remains unclear and warrants further investigation [137].

Nonetheless, evidence supporting caffeine’s ability to independently alter circadian clock timing or amplitude in the absence of light is scant [138]. For instance, studies in animals have demonstrated that under conditions of constant darkness, there were no discernible differences in rest-activity patterns between the subjects administered caffeine and those given a placebo [129]. Currently, investigations aimed at delineating purely circadian effects devoid of light stimuli necessitate experimentation under conditions of constant darkness, which poses logistical challenges in human studies. Consequently, the possibility remains that the observed effects of caffeine in humans, even under dim lighting conditions, may still stem from caffeine-induced enhancements in the circadian response to light.

### 6.4. Mechanisms of Caffeine on Strength and Power Exercise Performance

Future investigations into caffeine’s role in mitigating morning exercise performance decline also necessitate an examination of its effects on strength and power exercises. Scientific evidence indicates that caffeine enhances neuromuscular connections, optimizing muscle contractions and potentially increasing force production during strength training. Moreover, caffeine’s influence on the central nervous system can delay fatigue onset, aiding athletes in sustaining higher performance levels during high-intensity workouts [11].

Additionally, research suggests that caffeine supplementation may augment power output, particularly beneficial for activities requiring explosive movements. Furthermore, caffeine enhances focus and alertness, potentially improving technical execution and reducing injury risk during strength and power training. However, caution is warranted due to potential side effects such as elevated heart rate and sleep disturbances, emphasizing the need for careful dosage management. Further research is essential to fully understand caffeine’s mechanisms in counteracting exercise performance decline and optimizing training efficacy [139].

It is noteworthy that caffeine also plays a significant role in anaerobic performance. Studies have demonstrated that caffeine can enhance anaerobic performance in well-trained individuals and those engaging in intermittent physical activities, thanks to its synergistic effect with exercise. Particularly, caffeine exhibits high ergogenic potential in speed endurance exercises lasting 60 to 180 s. While research indicates caffeine’s impact on isometric maximal strength and preliminary evidence for enhancing muscular endurance in lower body musculature, its effects on isokinetic peak torque, single-repetition maximum torque, and muscular endurance in upper body musculature remain unknown [140].

The mechanism behind caffeine’s effects is believed to involve the enhancement of free fatty acid oxidation and glycogen sparing, possibly mediated by epinephrine-induced pathways [141]. However, this theory seems insufficient to explain improvements in anaerobic performance, which primarily relies on oxygen-independent metabolic pathways. Alternative mechanisms such as enhanced calcium mobilization and phosphodiesterase inhibition have been proposed, yet their significance at normal physiological doses remains uncertain [142].

Another hypothesis suggests that caffeine’s stimulation of the central nervous system plays a pivotal role. By antagonizing adenosine receptors, caffeine inhibits the negative effects of adenosine on neurotransmission, arousal, and pain perception. The analgesic effects of caffeine can reduce pain and perceived exertion during exercise, potentially leading to more sustainable and powerful muscle contractions [143]. Despite these insights, the exact mechanisms underlying caffeine’s effects on anaerobic performance remain elusive. Moreover, its role in alleviating morning performance declines requires further investigation to fully understand the intricate interplay between caffeine, exercise, and physiological responses.

### 6.5. Dosing Times of Caffeine in Different Dosage Forms

To enhance sports performance, the timing of caffeine intake varies depending on the dosage form. Oral tablets or capsules are typically consumed 30 to 60 min before exercise to ensure peak blood levels during the activity, optimizing the effects on endurance, strength, and cognitive function [144]. Liquid formulations, such as energy drinks or coffee, are also ingested around 30 to 60 minutes’ pre-workout, offering a quick boost in energy and focus [145]. Chewable tablets or gum, providing rapid caffeine absorption, serve as convenient options for immediate performance enhancement and can be taken shortly before exercise [146]. Extended-release formulations, less common but effective for sustained energy, are typically ingested 60 to 90 min before activity [147]. 

It is crucial for athletes to consider individual tolerance, sensitivity, and the duration of the activity when determining the optimal dosing time for caffeine in any form. Experimentation during training sessions can help identify the most effective timing and dosage. Additionally, moderation is key to avoid adverse effects such as jitters or gastrointestinal discomfort [148]. By aligning caffeine intake with the onset of exercise and tailoring it to personal needs and preferences, athletes can maximize the performance-enhancing benefits of caffeine across various dosage forms [5]. However, the extent to which these factors affect sports performance in practical applications, as well as the underlying mechanisms involved, remains largely unknown, warranting further investigation.

## 7. Conclusions

This review provides a comprehensive examination of the diverse ergogenic effects of caffeine on exercise capacity, particularly when consumed in the morning versus the evening. It suggests that caffeine’s ergogenic effects are more pronounced in the morning, likely attributable to the susceptibility of both regular exercise participants and elite athletes to declines in morning performance. Caffeine effectively mitigates this phenomenon, preventing declines and restoring performance to levels comparable to those observed during nighttime. The variability in findings across studies regarding caffeine’s ergogenic effects underscores the importance of specifying the timing of caffeine administration in future research endeavors.

Furthermore, several studies have indicated a close association between declines in morning performance during exercise and circadian rhythms, although the precise mechanisms through which circadian rhythms influence exercise performance remain unclear. While research has demonstrated caffeine’s ability to enhance performance through various mechanisms, including adenosine receptor blockade, increased muscle calcium release, and effects on catecholamines, caffeine also indirectly influences circadian rhythms by enhancing responsiveness to light-induced phase shifts. However, the specific pathways by which caffeine alleviates morning performance declines and subsequently enhances performance remain incompletely understood. Thus, further investigation is warranted to elucidate the underlying mechanisms driving caffeine’s ergogenic effects and its interaction with circadian rhythms.
